# The Association between Aortic Calcification and Fracture Risk in Postmenopausal Women in China: The Prospective Chongqing Osteoporosis Study

**DOI:** 10.1371/journal.pone.0093882

**Published:** 2014-05-09

**Authors:** Rui Zhou, Huadong Zhou, Min Cui, Lin Chen, Jianzhong Xu

**Affiliations:** 1 Department of Orthopedics, the Orthopedic Surgery Center of Chinese PLA, Southwest Hospital, Third Military Medical University, Chongqing, China; 2 Department of Neurology, Daping hospital, Third Military Medical University, Chongqing, China; 3 State Key Laboratory of Trauma, Burns and Combined Injury, Trauma Center, Institute of Surgery Research, Daping Hospital, Third Military Medical University, Chongqing, China; University of California, Los Angeles, United States of America

## Abstract

**Purpose:**

Fractures are associated with cardiovascular diseases in the elderly. The purpose of the present study was to investigate the association between aortic calcification (AC) and the risk of vertebral fractures in postmenopausal Chinese women.

**Methods:**

A prospective study with 5 years of follow-up in 1724 postmenopausal women (aged 50 years old and older) was conducted from July 2005 to June 2010. Dual energy X-ray absorptiometry was utilized to evaluate bone mineral density (BMD). Aortic calcification score (ACS) was determined by a semi-quantitative method and was further categorized into four groups. Cox proportional hazards models were established to assess the association between AC and the risk of vertebral fractures.

**Results:**

For subjects with AC, the incidence of vertebral fractures was higher than that of those without AC (p<0.01). After adjustment for age and other potential confounders, it was found that severe AC (G4, ACS>6; G3, ACS = 3–6) was associated with vertebral fractures. Severe AC (G4) was associated with non-vertebral fractures. There were higher risk for the vertebral fractures in two groups and higher risk for non-vertebral fractures in one group.

**Conclusions:**

The results of the current study indicate that severe AC is associated with a significantly increased risk of vertebral fractures and non-vertebral fractures in postmenopausal women in China.

## Introduction

Both fractures and cardiovascular diseases are important causes of morbidity and mortality in the elderly [1]–[3]. Postmenopausal women with cardiovascular diseases are at an increasing risk for fractures, independently of their age and cardiovascular risk profile. This fact suggests a possible association between cardiovascular diseases and fractures [4], [5]. This observation has been reported in studies involving subjects in both Asian and Western nations [6]–[8].

With regard to postmenopausal women, Svejme et al., in a 34-year prospective study, showed an association between cardiovascular disease and fractures [9]. Additionally, this study revealed associations between fractures and traditional risk factors for cardiovascular disease. The association between vascular calcification and fractures has been found to be significant after adjustments for age and other potential confounders [10]–[11].

China has the largest elderly population in the world, and as such, there have been increasing incidences of cardiovascular diseases and fractures [12]–[14]. However, it remains unclear whether AC is correlated with the risk of fractures in postmenopausal women in China. Thus, we conducted a 5-year prospective study to test the hypothesis that there would be a significant association between AC and the risk of vertebral fractures in postmenopausal women in the Chinese city of Chongqing.

## Methods

### Participants

The Institutional Review Board of Daping Hospital approved the present study, and all of the subjects provided informed consent. Chongqing is the largest municipality in Southwest China, with a population of 35 million. The subjects were sampled from three randomly selected communities within Chongqing.

Between January 1, 2005 and June 30, 2005, baseline screening was performed on subjects in the selected communities, in which 1993 postmenopausal women aged 50 years old and older were enrolled. The subjects excluded from this study were women with bone disorders, any rheumatologic or endocrine conditions, or any history of cancer within the previous 5 years. The subjects were interviewed in organized meetings at community centers. If subjects were absent from the meeting or were unable to attend due to physical disabilities, our staff performed the interviews at their homes. The subjects were followed up annually for 5 years from July 2005 to June 2010. After 5 years, 83 (4.2%) died, 129 (6.6%) withdrew participation, and 57 (2.9%) moved away during follow-up; thus, 1724 (86.5%) subjects completed the study.

### Data collection

The following baseline characteristics were collected during the enrollment stage.

#### (1) Basic data

Age, height, weight, body mass index (BMI), smoking and drinking status were recorded.

#### (2) Assessment of AC

A semi-quantitative method was used to rate aortic calcification [15]. Using baseline lateral radiographs of the lumbar spine, calcific deposits at the first four lumbar vertebrae of the abdominal aorta were identified in the posterior and anterior walls of the aorta, using the midpoint of the intervertebral space above and below the vertebrae as boundaries. Lesions were graded as follows: 0, no aortic calcific deposits; 1, small scattered calcific deposits filling less than one-third of the longitudinal wall of the aorta; 2, one-third or more, but less than two-thirds, of the longitudinal wall of the aorta calcified; and 3, two-thirds or more of the longitudinal wall of the aorta calcified. The severity scores for the eight segments of the posterior and anterior walls (0–3) were added to yield an AC score ranging from 0 to 24. Aortic calcifications scores (ACS) were divided into four groups: G1, ACS = 0; G2, ACS = 1-2; G3, ACS = 3-6; and G4, ACS>6. The readers assessing the radiographs read them independently and were not informed of the aim of the study. Inter-observer reproducibility was assessed by two radiologists. The intraclass correlation coefficient was 0.82.

#### (3) Fractures and falls

The incidence of vertebral fractures was determined using both morphometric criteria (McCloskey-Kanis method) [16] and a qualitative assessment (a vertebral body height reduction greater than 20%), determined by comparing the X-rays obtained at baseline and each year. Vertebral shape assessments included the ratios of anterior/posterior, central/posterior and posterior/predicted posterior vertebral heights from T4 to L5. The predicted posterior height was calculated from adjacent vertebrae. Assessment of all vertebral and non-vertebral fractures was conducted by comparing answers from the questionnaires with the original X-ray and medical reports. In addition, metacarpal, metatarsal or skull bone fractures were not included in the analysis. One reader assessed the fractures. The kappa score reflecting the intraobserver agreement was estimated to be 0.86 in the assessment of vertebral and non-vertebral fractures. Falls were determined by self-reporting.

#### (4) BMD

Dual energy X-ray absorptiometry (DXA, Prodigy fan beam densitometer, Lunar Corp, GE Medical System, Madison, WI, USA) was used to determine BMD. BMD of the L1-L4 vertebrae and hip (femoral neck, Ward triangle, trochanter and total hip) were measured at baseline.

#### (5) Clinical assessment

Medical history was collected from available medical records. The data included a history of coronary heart diseases, hypertension, diabetes mellitus and hypercholesterolemia. Blood pressure measurement and electrocardiography were performed on site. Diagnoses of diseases, including hypertension, diabetes mellitus, hypercholesterolemia, coronary heart diseases, and atrial fibrillation, were based on the International Classification of Diseases, 9th Revision (ICD-9).

#### (6) Blood measurements

Fasting blood samples (serum or plasma) were collected to measure glucose, total cholesterol, adiponectin, osteocalcin, leptin and 25(OH)D. Blood glucose levels were measured with a hexokinase enzyme reference method, and total cholesterol level was determined enzymatically with commercially available reagents (Roche Diagnostics, Mannheim, Germany). Adiponectin, osteocalcin, and leptin levels were determined with enzyme-linked immunosorbent assay (ELISA) kits, according to the manufacturer’s protocol (Shibayagi Co., Ltd., Gunma, Japan). Levels of 25(OH) D were measured by radioimmunoassay (^123^I RIA kit; DiaSorin, Stillwater, MN, USA).

### Follow-up

Written questionnaires and telephone interviews were used to collect follow-up information, such as fractures, changes in diseases and medicine usage, every year during the 5-year follow-up period.

### Statistical analysis

In univariate analysis, baseline variables between the subjects who developed aortic calcifications and those who did not were compared by using Pearson’s chi-square test, Fisher’s exact *t*-test or the Mann-Whitney *U*-test as deemed appropriate. In addition, the Cox proportional hazards model was used to assess the associations between aortic calcifications and vertebral fracture risk. The associations were first analyzed without adjustment for the other covariates. Then, the associations were further analyzed with adjustments for age (per 1 SD increase), fracture (yes vs. no), history of falls in the previous year (>1 versus 0-1), and other potential confounders. The statistical analyses were performed using SPSS (Statistical Package for the Social Sciences), version 15.0 software for Windows.

## Results

Table 1 summarizes the basic demographic characteristics and cardiovascular risk profiles of the two groups of subjects. A total of 1724 subjects were enrolled in this study. The distribution of ACS was skewed: 1051 subjects (60.9%) had no calcifications, 289 subjects (16.8%) had ACS of 1 and 2, 229 subjects (13.3%) had ACS between 3 and 6, and 155 subjects (9.0%) had ACS of>6 at baseline.

**Table 1:** Baseline characteristics of the subjects who developed aortic calcification and who did not in the study of 1724 women.

Factors	Total	No AC (score = 0)	AC (score≧1)	p value
	n = 1724	n = 1051	n = 673	
Age (yr)	69.3±9.3	68.3±8.5	72.4±7.9	<0.01
Weight (Kg)	53.1±12.4	52.7±11.7	54.4±11.3	0.073
BMI (Kg/ m2)	21.6±5.4	21.4±4.8	22.3±4.3	0.086
Vertebral BMD (g/cm2)	0.85± 0.01	0.89 ± 0.01	0.83± 0.01	< 0.01.
Hip BMD (g/cm^2^)	0.81± 0.01	0.86±0.01	0.78±0.01	< 0.01
History of two or more falls n (%)	297(17.2)	201(19.1)	96(14.3)	<0.01
Hypertension, n (%)	537(31.1)	291(27.7)	246(36.5)	<0.01
Diabetes, n (%)	383(22.2)	195(18.6)	188(28.0)	<0.01
Total cholesterol (mmol/L)	3.61±0.86	3.53±0.72	3.85±0.79	<0.01
Current smoking, n (%)	53(3.1)	29(2.8)	24(3.5)	<0.01
Current drinking, n (%)	28(1.6)	16(1.5)	12(1.8)	<0.01
Use of estrogen, n (%)	23(1.3)	12(1.1)	11(1.6)	0.384
Myocardial infarction, n (%)	65(3.8)	36(3.4)	29(4.3)	<0.01
Stroke, n (%)	29(1.7)	16(1.5)	13(1.9)	<0.01
Adiponectin (g/ml), mean ±SD	13.5±10.2	14.3±10.7	12.4±8.1	<0.01
Osteocalcin (mg/L)	26.6±14.5	26.1±13.7	27.3±12.4	0.066
Leptin (ng/ml), mean ±SD	21.5±18.6	21.3±13.4	21.8±15.7	0.480
25(OH)D (ng/mL)	14.4±6.9	14.4±5.1	14.3±6.8	0.728

AC = aortic calcification; BMD = bone mineral density.

The subjects with AC were older and had more frequent histories of two or more falls, hypertension, diabetes, current smoking, current drinking, myocardial infarction and stroke, higher Weight, BMI, total cholesterol than those without AC (p<0.01). It was found that the subjects with AC had significantly lower BMD in both the vertebrae and hips and adiponectin levels than those without AC (p<0.01).

### Association between ACS and fractures

**Figure 1:**
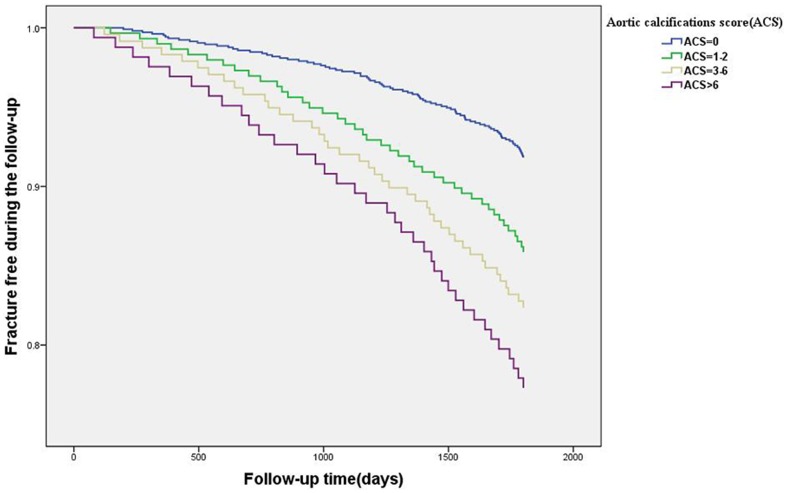
Fracture-free survival according to baseline ACS. Survival of women in this study cohort during over 5 years of follow-up, according to the ACS at baseline: ACS in G1 (0), G2 (1–2), G3 (3–6), and G4(>6). Fracture incidence was significantly higher in G2, G3, and G4, compared to G1, at the end of the 5 years of follow-up (p<0.001, respectively).

Among the 673 subjects with AC, a total of 122 (18.1%) of the subjects had fractures by the end of the follow-up, including 69 vertebral fractures and 53 non-vertebral fractures (33 hips, 7 distal radius, 5 ankles and 8 others). Figure 1 shows the fracture incidence every year over the 5-year follow-up period. For the subjects in the three higher ACS groups (G2, G3, and G4), the incidences of vertebral and non-vertebral fractures were higher than in the no ACS (G1) group (p<0.01). Table 2 shows the association of ACS with fracture incidence. Among the four groups of ACS, the incidences of vertebral and non-vertebral fractures in G1 (ACS = 0) was the lowest (4.5%, 3.9 %), and the incidences of vertebral and non-vertebral fractures in G4 (ACS>6) were the highest (12.2% and 9.7%, respectively).

**Table 2:** Relationship between ACS groups and the fractures.

Aortic calcification	Total	Vertebral fractures	Non- vertebral fractures		No Fracture
			hip fractures	other fractures	
	n = 1724	n = 116	n = 50	n = 44	n = 1514
G1 (ACS = 0), n (%)	1051	47 (4.5)	17 (1.6)	24 (2.3)	963 (91.6)
G2 (ACS 1–2), n (%)	289	26 (9.0)*	13 (4.2) *	7 (2.4)	243 (84.1)
G3 (ACS 3–6), n (%)	229	24 (10.4)*	12 (5.2) *	6 (2.6)	186 (81.3)
G4 (ACS >6), n (%)	155	19 (12.2)*	8 (5.2) *	7 (4.5)*	122 (78.8)
p trend		<0.01	<0.01	<0.01	

ACS  =  aortic calcifications score.

*p<0.01 vs G1.

### Association between age and fractures

**Figure 2:**
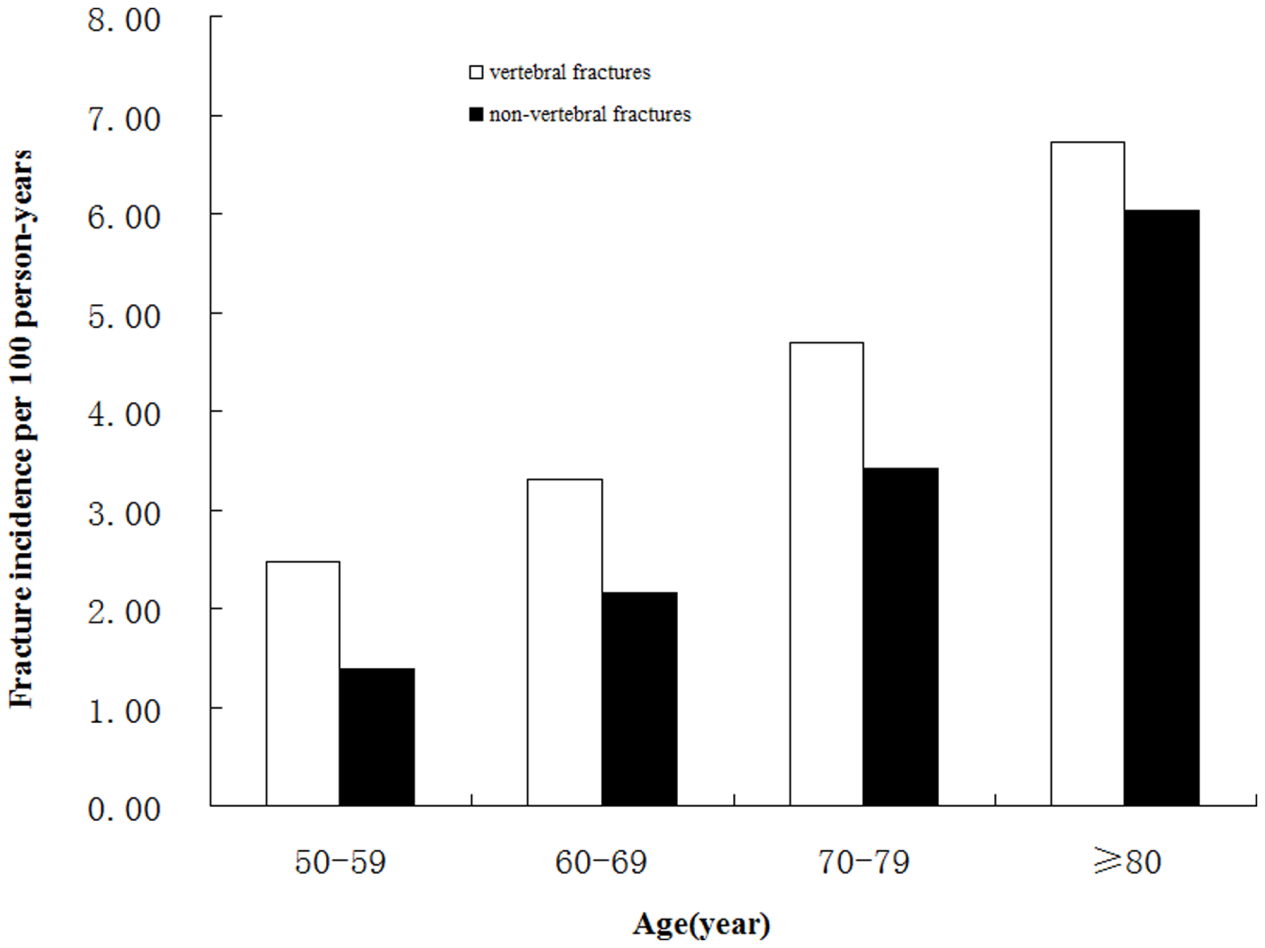
Incidence of vertebral and non-vertebral fractures per 100 person-years by age and site. Incidence of vertebral and non-vertebral fractures was significantly higher in the 60- to 69-year-old group, the 70- to 79-year-old group and the>80-year-old group compared to the 50- to 59-year-old group (p<0.001, respectively). In each group, the vertebral fracture prevalence was significantly higher than for non-vertebral fractures (p<0.001).

The association between age and fracture incidences in subjects with AC is shown in Figure 2. In the subjects 50 years old and older with AC, the incidences of vertebral and non-vertebral fractures increased with age. The lowest fracture incidence was observed in the subjects with no AC between 50 and 59 years old (vertebral fractures, 9.9%; non-vertebral fractures, 5.4%). The highest fracture incidence occurred in subjects with≥80 years old (vertebral fractures, 18.4%; non-vertebral fractures, 15.2%).

### Association between AC and risk of vertebral fractures

We established Cox proportional hazards models to obtain insights into the independent predictors of vertebral fracture risk, and the results are shown in Table 3. After adjustment for age, BMI, BMD, a history of two or more falls, current smoking, current drinking, previous fractures, hypertension, diabetes, total cholesterol, myocardial infarction, stroke, adiponectin, osteocalcin, leptin, and 25(OH)D, it was noteworthy that BMD, age, a history of two or more falls, and adiponectin were associated with vertebral fractures. Subjects in G3 (ACS = 3-6), G4 (ACS>6) had a higher risk of vertebral fractures than the subjects in G1 (ACS  = 0).

**Table 3:** Association between AC and vertebral fractures in Cox proportional hazard regression model.

Factors	Hazard ratio unadjusted		Hazard ratio adjusted*	
	HR	95% CI	HR	95% CI
ACS				
G1	1		1	
G2	1.54	0.86–3.21	1.67	0.93–3.46
G3	2.35	1.32–4.94	2.28	1.17–4.85
G4	3.91	1.81–7.92	3.15	1.35–6.18
Age (per 1 SD increase)	2.12	1.37–4.65	1.83	1.31–4.06
History of two or more falls	1.68	1.26–2.85	1.47	1.29–2.57
BMD (per 1 SD decrease)	3.37	1.99–6.08	2.92	1.67–5.34
Adiponectin (per 1 SD decrease)	1.24	1.23–2.34	1.23	1.19–2.32

BMD = bone mineral density, vertebral and hip BMD; ACS = aortic calcifications score; SD = standard deviation.

Adjusted for age, BMI, BMD, history of two or more falls, current smoking, current drinking, previous fractures, hypertension, diabetes, total cholesterol, myocardial infarction, stroke, adiponectin, osteocalcin, leptin and 25–(OH)D.

### Association between AC and risk of non-vertebral fractures

Table 4 shows the association between AC and risk of non-vertebral fractures. After adjustment for age, BMI, BMD, a history of two or more falls, current smoking, current drinking, previous fractures, hypertension, diabetes, total cholesterol, myocardial infarction, stroke, adiponectin, osteocalcin, leptin, and 25(OH)D, it was interesting that an ACS of G4, BMD, age and a history of two or more falls were associated with non-vertebral fractures.

**Table 4:** Association between AC and non-vertebral fractures in Cox proportional hazard regression model.

Factors	Hazard ratio unadjusted		Hazard ratio adjusted*	
	HR	95% CI	HR	95% CI
ACS				
G1	1		1	
G2	1.34	0.79–1.82	1.27	0.75–2.06
G3	1.95	0.96–2.43	1.68	0.93–2.31
G4	2.17	1.57–3.28	1.93	1.54–3.26
Age (per 1 SD increase)	1.92	1.15–2.61	1.87	1.21–2.98
History of two or more falls	2.94	1.75–4.32	2.76	1.68–4.53
BMD (per 1 SD decrease)	2.76	1.84–4.69	2.74	1.81–4.75
Adiponectin (per 1 SD decrease)	1.13	0.86–2.15	1.12	0.82–2.14

BMD = bone mineral density, vertebral and hip BMD; ACS = aortic calcifications score; SD = standard deviation.

Adjusted for age, BMI, BMD, history of two or more falls, current smoking, current drinking, previous fracture, hypertension, diabetes, total cholesterol, myocardial infarction, stroke, adiponectin, osteocalcin, leptin and 25(OH)D.

## Discussion

Our study is the first report indicating an association of AC with a risk of future fractures in postmenopausal women in China. Severe AC (ACS>6, G4) was associated with a 3-fold greater risk of vertebral fractures. The results also revealed that adiponectin was an independent risk factor associated with vertebral fractures in postmenopausal women. These results suggest a relationship between atherosclerotic vascular disease and bone metabolism.

This study showed that an association between AC and vertebral fractures has been demonstrated in Asian women [17]. After adjusting for confounding factors, patients with AC had more than a 3-fold increased risk of vertebral fractures. This result indicated that AC was significantly associated with the prevalence of vertebral fractures in Asian women. El Maghraoui et al. [18] reported that extended abdominal aortic calcification is independently associated with prevalent vertebral fractures regardless of age, BMI, history of fractures, and BMD in post-menopausal women. In another study, there were no significant differences in the frequency of vertebral fractures between subjects with Abdominal AC and those without Abdominal AC (p > 0.05) [19].

More recently, Wang et al. [20] did not find an association between fractures and the prevalence or severity of abdominal AC in a 5-year study of 1471 healthy postmenopausal women. During follow-up, the women with baseline abdominal AC did not have an increased number of incident osteoporotic fractures (114 versus 99, p = 0.23) or total fractures (140 versus 130, p = 0.41) compared with women without baseline abdominal AC; however, the AC group did experience an increase in the number of incident hip fractures (14 versus 9, p = 0.39). A prospective epidemiological study that included 2662 postmenopausal women with a mean age of 65.0±7.1 years old at baseline confirmed the relationship between AC and hip fractures [21]. In a multivariate logistic regression model, age, body mass index and the severity of AC were independent predictors of hip fractures.

Adiponectin has been implicated in bone metabolism and is negatively associated with BMD. Several studies have suggested that higher levels of serum adiponectin are associated with incident fractures in elderly men [22], [23]. Barbour and colleagues [24] found a significant association between serum adiponectin and all fractures in men but not in women. In our study, we found that plasma adiponectin levels were independently associated with an increased risk of vertebral fractures in postmenopausal women. Thus far, no other studies have shown a relationship between adiponectin and fracture risk in women. However, one study suggested that the association between adiponectin and bone might be influenced by sex hormones [25].

The presence of the bone proteins osteopontin, osteocalcin, and BMP2 in calcified vascular lesions suggests that osteogenic mechanisms might play a role in vascular calcification [26]–[28]. In fact, vascular smooth cells derived from large- and medium-caliber arteries underwent calcification under several in vitro experimental conditions [29]. A recent study found that bovine aortic smooth muscle cells lost their lineage markers under calcifying conditions, and meanwhile, the cells gained an osteogenic phenotype as indicating by an increase in expression of osteopontin, osteocalcin, and core binding factor alphal 1 (Cbfa1) [30]. This study found that AC was associated with a similar loss in smooth muscle markers and a gain of osteopontin and Cbfa1 expression. These data showed a novel association of vascular calcification with smooth muscle cell phenotypic transition.

There were several limitations to the present study. First, information on impaired vitamin K status, which might be associated with both atherosclerosis and BMD [31], was not available for this study. Second, secondary hyperparathyroidism, which can be induced by vitamin D deficiency in the elderly, might be associated with bone loss, as well as with soft-tissue calcium deposition [32]. Third, the association between AC and non-vertebral fractures (hip, distal radius, ankle and others) observed in this study was limited by the small sample size.

In conclusion, the present study revealed that severe AC and high plasma adiponectin levels were independent predictors of vertebral fractures in postmenopausal women in China. Thus, mitigating vascular risk factors could reduce fracture incidence. In addition, our study showed an association between adiponectin and fractures. Thus, investigations into the roles of sex hormones in adiponectin could prove illuminating.
